# Prospective subtyping identifies an association between high pretreatment levels of DEspR^+^ neutrophil extracellular trap–forming neutrophils and microvesicles during sterile inflammation with poor survival in patients with metastatic non-small cell lung cancer receiving first-line combination chemo-immunotherapy

**DOI:** 10.3389/fimmu.2026.1849858

**Published:** 2026-08-05

**Authors:** Victoria L. M. Herrera, Kiana Mahdaviani, Jacqueline Choi, Sin-Han Chen, Eliot Gunn, Kurtis Stadnicki, Anais Mortazavi-Zadeh, Judith J. Lok, Umit Tapan, Nelson Ruiz-Opazo

**Affiliations:** 1 Whitaker Cardiovascular Institute, Chobanian & Avedisian School of Medicine, Boston University, Boston, MA, United States; 2 Department of Medicine, Boston Medical Center, Boston, MA, United States; 3 Department of Mathematics and Statistics, Boston University, Boston, MA, United States

**Keywords:** chemo-immunotherapy, DEspR, metastatic NSCLC, microvesicles, NET-formation, neutrophil extracellular traps, neutrophil subtypes, PDL1+ neutrophils

## Abstract

**Introduction:**

High neutrophil-lymphocyte ratio (NLR) and neutrophil extracellular traps (NETs) are associated with poor overall survival in many cancers but are currently remain without safe effective therapy until subtype elucidation identifies a targetable molecular subtype whose inhibition will not compromise homeostatic pathogen defense. We tested the hypothesis that increased circulating NET-forming neutrophils (NET^+^Ns) expressing the cell-surface dual endothelin-1/signal receptor (DEspR) comprise a subtype that predicts poor survival outcomes in patients with metastatic non-small cell lung cancer (mNSCLC) receiving first-line chemotherapy-immunotherapy (chemo-IO), because peripheral levels of DEspR^+^ neutrophils and NET^+^Ns are increased in neutrophil-mediated secondary tissue injury during sterile inflammation across different diseases in which high NLR is associated with disease progression or exacerbation.

**Methods:**

In a prospective observational study, we subtyped neutrophils by flow cytometry and NET^+^Ns by immunofluorescence cytology (IFC) confocal microscopy using fresh whole blood samples from 10 patients with mNSCLC at baseline and 6 weeks after the first dose of chemo-IO. We performed Kaplan–Meier survival analysis and Cox regression with Firth’s penalty for 2-year overall survival (OS) and restricted mean survival time (RMST) analyses for 1-year OS.

**Results:**

In 10 patients with mNSCLC, immunosuppressive PDL1^+^DEspR^+^ neutrophils and monocytes were increased at pretreatment baseline compared with normal levels. IFC detected increased circulating DEspR^+^citH3^+^[NET^+^Ns] and microvesicles (MVs), the majority of which were also PDL1^+^, and persisted 6 weeks after the first dose of chemo-IO. IFC of buffy coat smears detected DEspR^+^[NET^+^Ns] complexed with DEspR^+^EpCAM^+^ circulating tumor cells (CTCs). Concordant findings from Kaplan-Meier survival analysis and univariate Cox regression with Firth's penalty for 2-year overall survival (OS), and RMST for 1-year OS identified significant association between high baseline levels of DEspR+[NET+N] subtpe and overall survival respectively.

**Conclusions:**

These data identify the DEspR^+^ subtype of citH3^+^/PDL1^+^[NET^+^Ns] and MVs whose respective levels correlate with decreased 1-year and 2-year OS in patients with mNSCLC receiving first-line chemo-IO. Visualization of NET^+^N intercellular complexes in circulation depicts ongoing systemic inflammatory-immunosuppressive microenvironment in mNSCLC. As DEspR is cell-surface accessible and targetable, these data collectively provide a basis for further confirmatory research to evaluate DEspR as a potential actionable biomarker in larger and independent mNSCLC cohorts.

## Introduction

Immune checkpoint inhibitor (ICI) therapies have demonstrated breakthrough efficacy by improving outcomes for patients with non-small cell lung cancer (NSCLC) compared with standard of care chemotherapy across all stages (1). While primary and acquired resistance to monotherapy and combination therapies continue to occur (2, 3), greater efficacy and lower rates of primary resistance have been reported with immune-checkpoint inhibitor plus chemotherapy-immunotherapy (chemo-IO) in metastatic non-small cell lung cancer (mNSCLC); therefore, this regimen has been approved as first-line therapy (2, 4). For mNSCLC with programmed cell death ligand-1 tumor proportion score (PDL1-TPS) < 1%, improved overall survival (OS) and progression-free survival (PFS) have also been observed (5). This efficacy is attributed to ICI-mediated enhancement of immunogenic tumor cell death and elimination of neutrophil-mediated immunosuppression via chemotherapy-induced neutropenia and the release of tumor antigens following tumor cell killing (6, 7). However, despite a two-fold decrease in primary resistance (defined as no response by 6 months) with first-line chemo-IO compared with IO-monotherapy, primary resistance still occurs in 10% of patients with mNSCLC (2).

Cumulative data implicate neutrophils and neutrophil extracellular traps (NETs) in therapy resistance and poor outcomes across multiple cancer types (8–10). As neutrophils are the majority intratumoral immune cell population in NSCLC (8), intratumoral neutrophils disrupt the “permissive microenvironment” required for the execution of tumoral immunogenic cell death (ICD) (11). Neutrophilic degradative proteases and myeloperoxidase impair the functions of cytotoxic T cells and natural killer (NK) cells, and NET DNA webs act as shields for tumor cells, blocking tumor cell-T cell synapses required for immune cell death (8–10). However, therapeutic inhibition of neutrophils and NETs remains highly challenging as neutrophils and NETs are the first-line defenders against infections, resulting in an unfavorable efficacy/toxicity ratio in clinical trials of diseases with neutrophil-driven pathologies. Advances in understanding neutrophil subtype heterogeneity provide a path forward but require identification of subtype-specific markers to distinguish homeostatic neutrophils involved in pathogen/tumor cell-killing from immunosuppressive/tumor-protecting and NET-forming neutrophil (NET^+^N) subsets (12).

Additionally, the emerging importance of systemic immunity in cancer and responses to immunotherapy (13) highlights the clinical relevance and need to study circulating neutrophils in addition to tumor-associated neutrophils. As significant human-mouse/animal species differences in neutrophil biology have emerged (14), translational research using cancer patient samples to determine the roles of neutrophils and NET^+^Ns (10) in systemic immunosuppression (15) are expected to provide critical human-specific insights (16).

Our work on neutrophil and NET^+^N subset analyses in acute and chronic diseases exacerbated by dysregulated neutrophils has identified the DEspR^+^CD11b^+^ neutrophil subset in humans (17–19). This DEspR^+^CD11b^+^ neutrophil subset exhibits increased lifespan and adhesion *in vitro* (20), and comprises the majority of circulating NET^+^Ns with release of NET-associated microvesicles (netMVs; hereafter referred to as MVs) during sterile inflammation in non-malignant conditions (18, 19). As these neutrophil properties are reported in immunosuppressive myeloid cells, we explored 1] the co-expression and differential levels of PDL1 and DEspR in circulating neutrophils and NET^+^Ns in patients with mNSCLC, 2] potential role as cancer stem cell protective escorts, 3] changes, if any, after first-line chemo-IO, and 4] comparative relationship(s) to clinical outcomes, if any.

## Methods

### Prospective pilot study of mNSCLC on first-line chemo-IO

We conducted a prospective pilot observational study of patients with mNSCLC treated at Boston Medical Center between June 2022 and August 2024. After obtaining informed consent in accordance with the IRB-approved protocol, blood samples were collected before the first dose of chemo-IO (baseline) and 6 weeks after dose 1. Patients were monitored until August 2025, with IRB-approved follow-up of up to 24 months.

### Detection of DEspR± CD11b ± PDL1± neutrophils in mNSCLC

To define neutrophil subtypes, we used a multiplex flow cytometry panel to evaluate DEspR ± CD11b ± PDL1± neutrophils in blood samples obtained from patients with mNSCLC within 1 h of collection and without prior cell isolation, to avoid a time-dependent increase in neutrophil activation and DEspR+ neutrophil expression *ex vivo* as described previously (17). Binding of antibodies was performed at 4 °C in fresh HBSS containing 2% FBS (without fixative, unlike commercial buffers), followed by light fixation in 1% paraformaldehyde (PFA) after binding and before red blood cell (RBC) lysis. Same-day flow cytometry was performed using an Aurora spectral cytometer. Antibodies used for flow cytometry were as follows: anti-DEspR antibody (hu6g8 with an IgG4[S228P] backbone) conjugated to fluorophore AF-647 in-house (final concentration in 200 μL: 10 μg/ml); CD11b-AF488 (3.12 μg/mL) (BioLegend, 101217); PDL1 (0.5 μg/mL) (Abcam, ab228413) conjugated in-house with AF488; double strand DNA-AF488 (10 μg/mL) (NeoBiotechnologies, MSM2-946-P1ABX). In-house fluorophore conjugations were performed using AF647 and AF488 labeling kits (Invitrogen A88068 AF647 and A88062 AF488, respectively).

### Direct visualization of NET formation in the circulation in mNSCLC

To determine circulating levels of NET-forming neutrophils, we analyzed peripheral blood samples without neutrophil isolation, enrichment, or culture, as neutrophils are highly reactive to microenvironment changes. Peripheral EDTA-anticoagulated blood samples were prepared and fixed for immunofluorescence cytology (IFC) <1 hour from blood draw, with 5 μL per slide, as described previously by our group (17). Separately, 5 μL of buffy coat isolated from 10 mL of EDTA-anticoagulated whole blood was prepared on PLUS slides, fixed in –20 °C methanol within 1 h from blood collection, and stored at –20 °C until IF-staining. We performed two-antibody probe IFC with different antibody probes paired with anti-DEspR antibody. All antibody pairs were incubated for 2.5 h at room temperature after a 2-hour blocking step at 4 °C: citrullinated histone3 (50 μg/ml) (citH3 Arg2, Arg8, Arg17, AbboMax 630-180ABBOMAX) conjugated in-house with AF488, conjugated myeloperoxidase-AF488 (1:500 dilution) (MPO-AF488, Abcam AB225474-1002), programmed death ligand-1 (PDL1) conjugated in-house, conjugated Mcl1-AF488 (50 μg/ml) (G7)-AF488 (SCBT sc74437), epithelial cell adhesion molecule (conjugated EpCAM-AF 488 (Abcam ab237395 [EPR20532-225])), and humanized anti-DEspR mab (50 μg/ml) (hu6g8-AF568) in-house conjugation as the common partner to all AF488 labeled antibodies.

Microscopy was performed using Zeiss Axioskopf 100X-oil objective (1,000x magnification) and confocal fluorescence photomicroscopy with 0.25 micron Z-steps and extended depth of field z-stack imaging (Nikon BioImaging Laboratory; 60x-oil objective or 600x magnification). Quantitation was performed in six high-power fields (HPFs) by two observers blinded to outcomes. HPFs focused on regions of interest (ROIs) with NET^+^Ns identified by automated full slide scan at 200x magnification for all patient cytology slides. The top six HPFs with the most NET+Ns were imaged by confocal photomicroscopy. whileAreas without NET+Ns were also documented to ascertain that detection of NET^+^N extruded DNA was not due to IF-cytology slide preparation or staining.

### Statistical analyses

Kaplan–Meier (K–M) curve analysis was performed using PRISM GraphPad 10.4, with log-rank tests applied to assess differences between survival curves. Cutoff thresholds for predictors were based on medians for a clinically relevant binary predictor-based approach. Univariate Cox regression was performed on data with the 2-year OS using Firth’s penalized likelihood method to reduce small sample bias and the Benjamini–Hochberg procedure to reduce false discovery rates. Analyses were implemented in R version 4.5.0 within the RStudio environment version 2024.12.1 (21, 22).

Restricted mean survival time (RMST) analyses were performed using the *survRM2* package in R (23, 24). Univariate linear regression analyses were performed on data with 1-year OS relevant to the 1-year survival rate of 31% observed in patients with mNSCLC (25) and relevant to restricted mean survival time analysis in oncology (26). Only data that passed normality testing and had statistical power > 0.8 were included in the linear regression analyses, which were performed using SigmaStat 4.0 and R.

### Immunofluorescence-staining of NSCLC adenocarcinoma tumor microarray

To analyze PDL1 and DEspR expression in NSCLC, we performed immunofluorescence (IF)-staining of a lung cancer tumor microarray focused on NSCLC adenocarcinoma primary lung tumors and corresponding lymph node metastases (US Biomax LC814a). Double immunofluorescence staining was performed using labeled PDL1-AF488 and DEspR-AF58 antibodies, essentially as described previously (17). Extended depth of focus (EDF) confocal composite of 0.25 μm z-stacks were obtained from each tumor and lymph node core using an automated confocal fluorescence microscopy system (Nikon BioImaging Laboratory).

### Detection of DEspR-specific spliced, Trp #14 codon transcript in RNA-seq cancer files

To overcome the difficulty of detecting DEspR-strand specific transcripts distinguished from the overlapping transcript (FBXw7-exon 7) on DEspR’s antisense strand in most RNA-seq files that are non-strand-specific, analysis was performed by querying the DEspR-specific transcript sequence that is comprised of spliced DEspR exon-1 and exon-2, which contains trp#14 codon sequence rather than the stop codon reported for FBXW7-AS1 (**Supplementary Figure 3A**). This sequence was investigated stepwise using sample channel staging as follows: open access RNA-seq files, confirmed human-specific, transcripts bearing the Trp#14 sequence (*rather than stop codon in FBXw7-AS1)*, and DEspR-specific spliced transcript that is distinguished from exon-7 of the antisense transcript FBX7 (**Supplementary Figures 3A, B**).

## Results

### Patient demographics

In this prospective observational study, we enrolled a total of 10 patients with mNSCLC treated with first-line chemo-IO (**Supplementary Table 1**). Chemo-IO regimens comprised of 1] pembrolizumab, carboplatin, and pemetrexed for stage IV adenocarcinoma (8/10); 2] pembrolizumab, carboplatin, and nab-paclitaxel for stage IV squamous cell carcinoma (1/10); or 3] durvalumab, carboplatin, and paclitaxel for stage III-A adenocarcinoma (1/10). The % tumor proportion score (%TPS) for PDL1 expression ranged from 0% to 95%, the neutrophil-to-lymphocyte ratio (NLR) ranged from 0.9 to 9.76, and ECOG performance status ranged from 0 to 2. There were no patients with a complete response.

### Comparison of NLR and DEspR^±^ CD11b^±^ PDL1^±^ neutrophil subtypes

**Figure 1:**
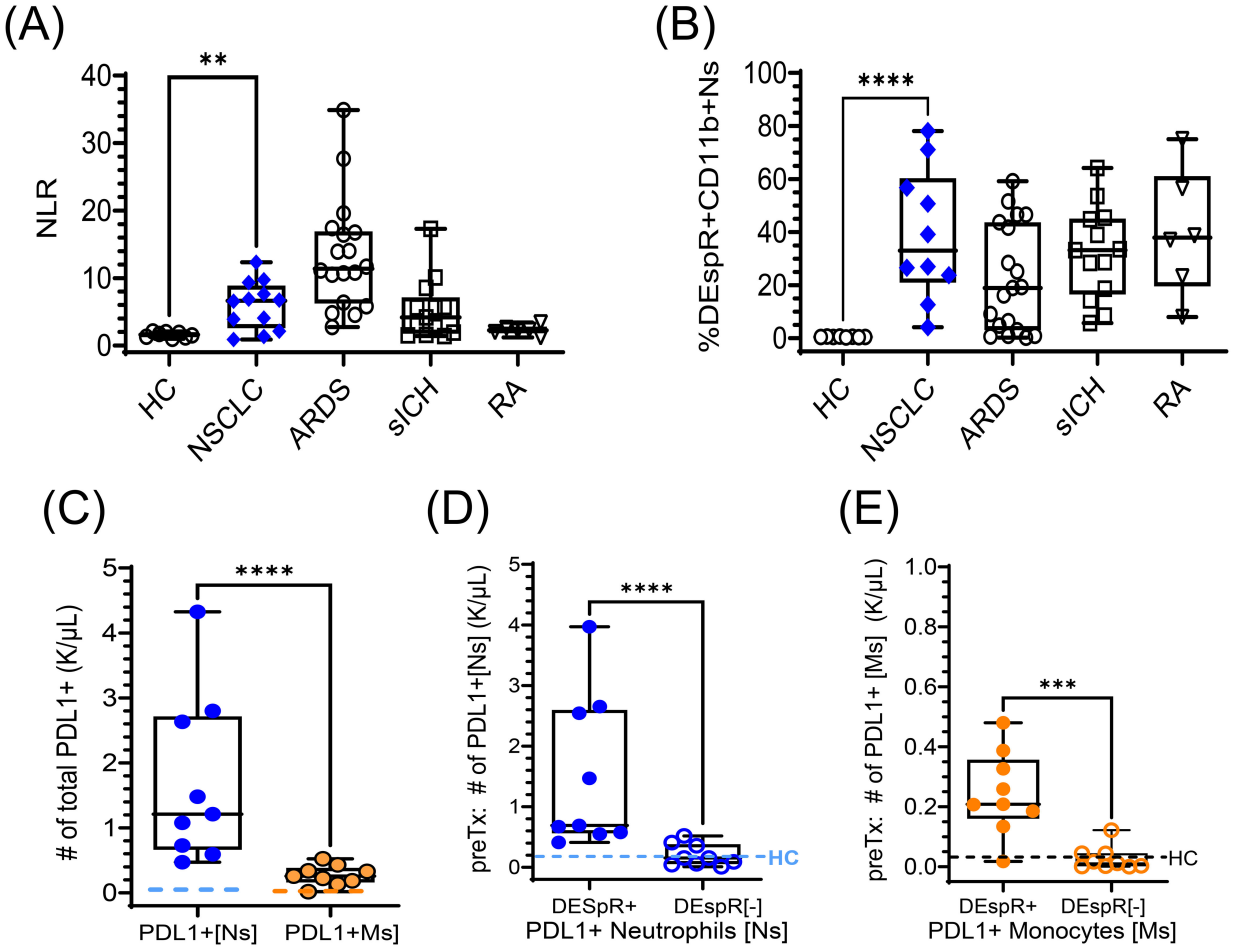
Comparison of NLR, proportion (%) of DEspR+CD11b+ neutrophils [Ns], and number (K/μL) of DEspR+PDL11+ *vs* DEspR[-]PDL1+ neutrophils [Ns] and monocytes [Ms] in mNSCLC patients. **(A)** Graph comparing baseline NLR in metastatic NSCLC patients (NSCLC, blue diamond symbols) with prior reports (open symbols) in healthy controls and DEspR[-] COVID patients (HC), and in patients with ARDS (acute respiratory distress syndrome Berlin criteria), sICH (spontaneous intracerebral hemorrhage), and RA (rheumatoid arthritis at exacerbation) (16–18, 27). Two-tailed Mann–Whitney non-parametric test between mNSCLC and HC, **p < 0.0055. **(B)** Graph comparing proportion (%) of DEspR+CD11b+ neutrophils (Ns) detected on flow cytometry at baseline pretreatment for mNSCLC patients compared to the HC group and other published patient studies (open symbols) as in graph **(A)** Two-tailed Mann–Whitney non-parametric test, mNSCLC vs HC, ****p < 0.0001. **(C)** Baseline (pretreatment or preTx) number (#) of DEspR+ *vs* DEspR[-] PDL1+ neutrophils and monocytes in mNSCLC. HC levels (aqua - - -) of total PDL1+ Ns; HC levels (orange - - -) of total PDL1+ [Ms] as reported by others (25, 26). **(D)** Baseline levels of PDL1+ neutrophil [Ns] subsets distinguished by cell surface expression of DEspR+ vs DEspR[-] by flow cytometry. HC, healthy control level (aqua -- -). **(E)** Baseline levels of PDL1+ monocyte [Ms] subsets distinguished by cell surface expression of DEspR+ vs DEspR[-] by flow cytometry. HC, healthy control levels. Mann–Whitney rank order test: ***p < 0.001; ****p < 0.0001.

To assess the role of neutrophils and NET^+^Ns, we first assessed NLR in patients with mNSCLC and compared it with NLR levels in non-malignant conditions in which NLR correlates with poor outcome measures. Comparative analysis demonstrated mild-to-moderate elevation of NLR in mNSCLC at baseline, in contrast to higher NLR levels in acute hyperinflammatory states, such as acute respiratory distress syndrome (ARDS) and spontaneous intracerebral hemorrhage (sICH) (**Figure 1A**).

In contrast, the proportion (%) of DEspR^+^CD11b^+^ neutrophil subtype was relatively high in mNSCLC compared with ARDS, sICH, and healthy controls (17, 18) (**Figure 1B**), consistent with the emerging importance of systemic inflammation in cancer (28).

Next, we assessed immunosuppressive PDL1^+^ myeloid cells. Compared with published levels in healthy control (29, 30), PDL1^+^ neutrophils and monocytes were increased in all mNSCLC patients at baseline, with PDL1^+^ neutrophils outnumbering PDL1+ monocytes (**Figure 1C**). Subtype analysis demonstrated that the majority of PDL1^+^ neutrophils and monocytes were DEspR^+^ (**Figures 1D, E**), indicating an apoptosis resistance advantage associated with DEspR^+^ expression, as previously observed *ex vivo* in ARDS patients (17) and in rhesus macaque neutrophils (20).

### Detection of NET formation in the circulation at baseline by direct visualization

**Figure 2:**
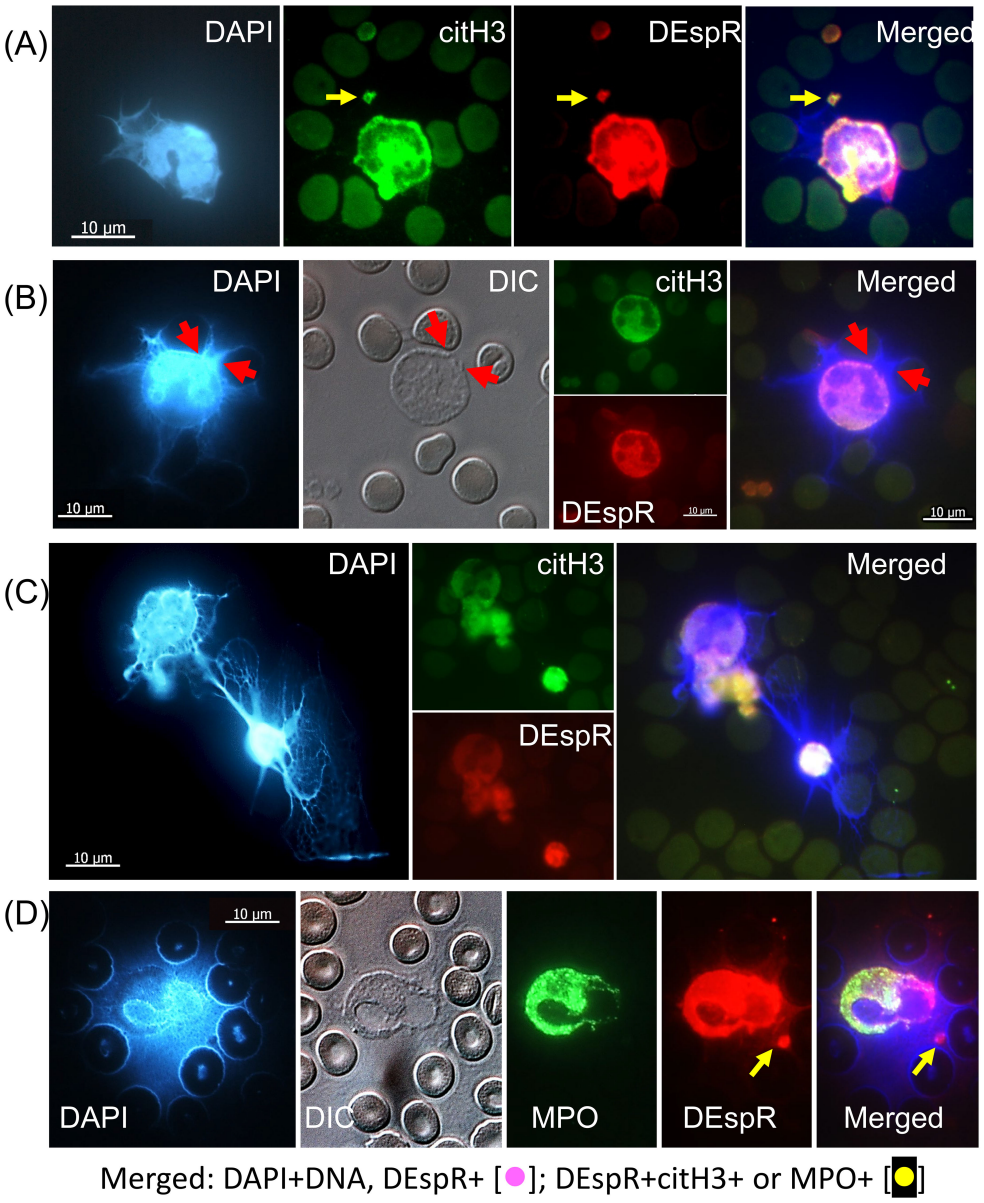
Representative images of circulating [NET^+^Ns] in peripheral blood samples from mNSCLC patients expressing DEspR and exhibiting NET-hallmarks. **(A)** Multiple high-resolution panels showing [NET^+^N] immunostained with DAPI+ DNA in decondensed nucleus and extruded DNA, citH3+ expression in the nucleus, cytoplasm, along the cell membrane, and in a released microvesicle (netMV) (yellow ➔), DEspR+ expression similar to citH3 subcellular locations and in released microvesicle (➔), Merged showing co-expression sites. Background autofluorescence (dull green) of red blood cells (RBCs) is shown. **(B)** Panels show NET^+^N with DAPI+ DNA extruding through a break (➔←) in the cell membrane, which is confirmed on the DIC image; citH3+ and DEspR+ expression, and merged images. **(C)** Representative image of two DAPI+, citH3+, DEspR+ [NET^+^Ns] forming a doublet, and varying degrees of DNA-web extrusion and cell dysmorphology. **(D)** Representative images of [NET^+^N]: DAPI+ decondensed nucleus with extruded DAPI+ DNA-web wrapping around RBCs appearing as “ringlets”; DIC image of [NET^+^N] with decondensed nucleus approximating cell membrane as described for NET-DNA extrusion; MPO+ and DEspR+ expression, Merged image; (yellow ➔) DEspR+DAPI+ netMV, bar = 10 microns.

Direct visualization via IFC enables molecular subtyping, cell morphology, and interaction analyses of NET^+^Ns in peripheral blood, providing insights into mNSCLC that are not obtainable using non-visualizing detection methodologies (31). IFC detected DEspR^+^ PDL1^+^ NET^+^N subtype in peripheral blood, which exhibited NET-formation hallmarks, including citrullinated histone-3 (citH3)^+^DNA, microvesicle release (**Figure 2A**), and nuclear decondensation of the still recognizable polylobulated nucleus that abuts cell membrane breaks showing DNA extrusion (**Figure 2B**).

Consistent with NET functionality (32), inter[NET^+^N] interactions or contacts were observed (**Figure 2C**). Cytoplasmic DNA was citH3^+^, a hallmark for NETs, with rimming by cell membrane (**Figures 2A, B**). However, the majority of extruded DNA appeared citH3^[-]^ or below citH3 IFC-detection levels (**Figures 2A, B**), although some were citH3^+^ (**Figure 2C**).

**Figure 3:**
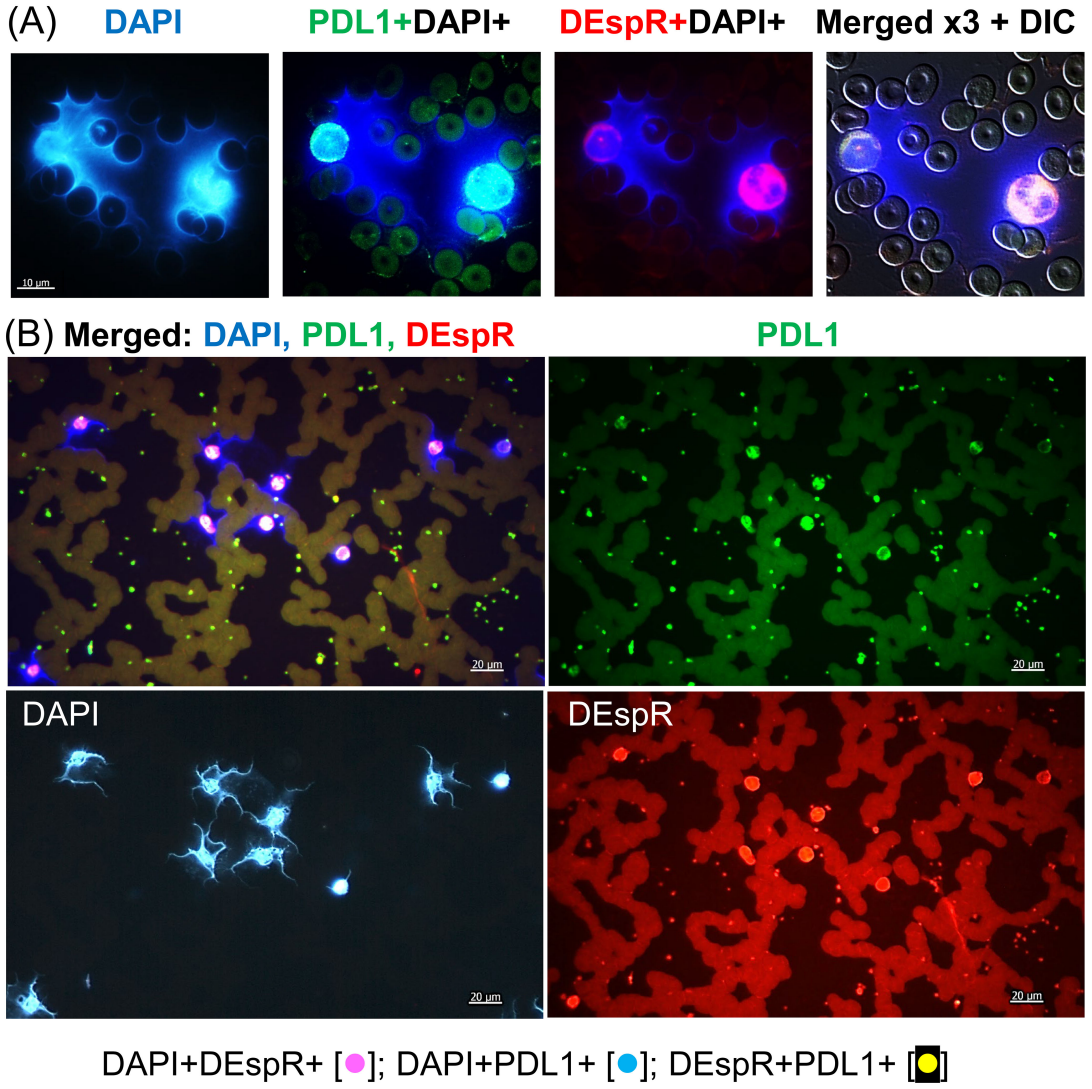
Representative images of DEspR+PDL1+ [NET^+^Ns] and NET-associated microvesicles (netMVs). **(A)** DEspR+PDL1+ [NET^+^Ns] with extruded DAPI+DNA wrapped around adjacent RBCs, and different stages of nuclear decondensation; PDL1+DAPI+ image; DEspR+DAPI+ image; merged x3 +DIC, DAPI+DEspR+PDL1+ merged image with differential interference contrast (DIC) confirming cell morphology of [NET^+^Ns] with extruded DNA wrapped around RBCs; bar = 10 microns. **(B)** Representative images of [NET^+^Ns] surrounded by microvesicles (netMVs) showing merged co-expression of DAPI, PDL1, DEspR in [NET^+^Ns] and netMVs; PDL1+ single immunostaining of PDL1+ [NET+Ns] and netMVs; DAPI+ staining of [NET^+^Ns] with extruded DNA-web; some cells are without extruded DNA, ascertaining specificity of [NET^+^N] identification; and DEspR+ [NET^+^Ns] and netMVs. RBC autofluorescence overexposed to delineate relative location to RBCs in blood smears; bar = 20 microns.

Another NET-formation hallmark, MPO^+^ nuclear expression was observed, along with nuclear decondensation and swelling and microvesicle release (**Figure 2D**). Interestingly, NET DNA wrapped around adjacent intact RBCs (**Figure 2D**), a finding consistent with immune-evasion caused by NETs acting as biophysical shields that interfere with cell-cell synapse formation required for tumor cell killing by killer T cells and natural killer (NK) cells. Across multiple representative NET^+^N images shown, differences in the extent of nuclear decondensation, DNA extrusion, and nuclear and cell morphology indicate non-synchronous NET-formation among circulating NET^+^Ns in the mNSCLC intravascular milieu (**Figures 2** and **3**).

Importantly, during our analysis, we confirmed that the detection of NET^+^Ns and the observed cell-cell complexes were not due to artifacts of whole blood specimen formation or IF-staining, as there are many areas with no NET^+^Ns and no cell-cell complexes (**Supplementary Figures 1A, B**).

### Immunosuppressive PDL1^+^DEspR^+^ [NET^+^N] subset and MVs at baseline and on chemo-IO

Next, we investigated whether NET^+^Ns also express PDL1, a molecular marker for immunosuppressive neutrophils, and DEspR, a molecular marker previously associated with increased lifespan in both neutrophils (20) and cancer stem cells (33). As shown in **Figure 3A**, PDL1^+^DEspR^+^ [NET^+^Ns] were detected at different stages of NET formation and, surprisingly, with the release of PDL1^+^DEspR^+^ MVs (**Figure 3B**).

**Figure 4:**
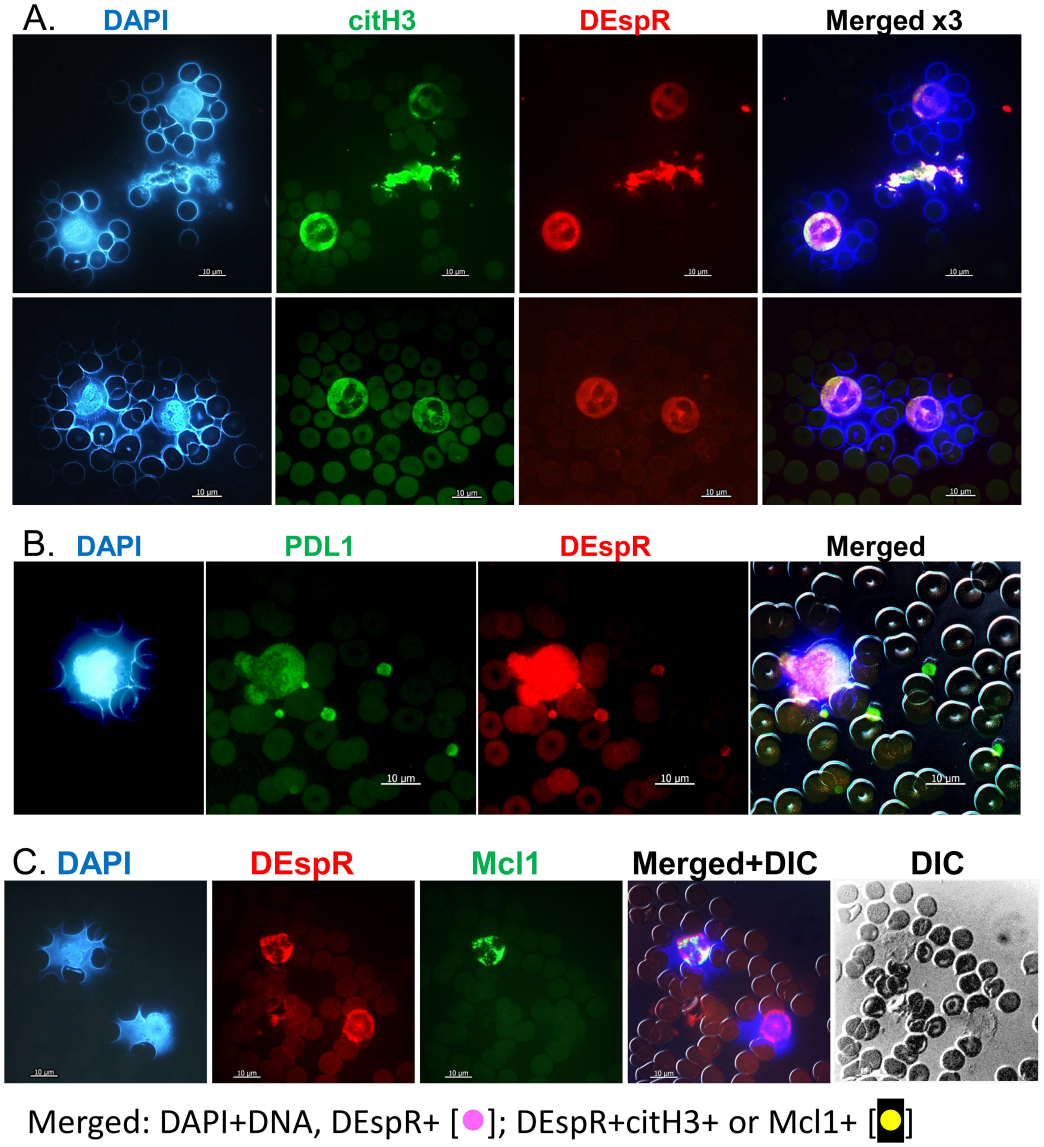
Representative images of DEspR+PDL1+ [NET^+^Ns] 6 weeks after the first dose of chemo-IO. **(A)** DEspR+citH3+ [NET^+^Ns] with DAPI+ DNA strands encircling RBCs forming “ringlets” concordant with positively charged citH3+ DNA, citH3, and DEspR+ co-staining showing different stages of NET formation in the circulation, merged x 3: DAPI, citH3, and DEspR. **(B)** DEspR+PDL1+ [NET^+^Ns]: DAPI+ DNA in a decondensed nucleus and extruded DNA webs, PDL1+ [NET^+^N] releasing netMVs; DEspR+ [NET^+^N] and microvesicles. **(C)** Representative image showing a DEspR+Mcl1+ [NET^+^N] with a still recognizable polylobulated decondensed nucleus, and a DEspR+Mcl1[-] NET^+^N with a decondensed ring-like nucleus; Merged+DIC image: DAPI, DEspR+, Mcl1+; DIC: differential interference contrast showing relatively intact cell membrane. Bar = 10 microns. .

To determine the impact of chemo-IO on NET^+^Ns, we examined NET^+^Ns 6 weeks after the first dose of chemo-IO using the same experimental protocol. IFC detected persistence of DEspR^+^ [NET^+^Ns], confirmed by detection of citH3^+^, a hallmark of NET formation, after the first dose of chemo-IO. As observed at baseline, DEspR^+^ [NET^+^Ns] exhibited DAPI^+^ NET-DNA wrapped around adjacent RBCs (**Figures 4A, C**), release of DEspR^+^citH3^+^ MVs (**Figure 4A**), which were also DEspR^+^PDL1^+^ MVs (**Figure 4B**), and in some MVs, DNA+ (**Supplementary Figure 2**).

Notably, NET^+^Ns were DEspR^+^Mcl1^+^, a pro-survival protein required for neutrophil survival (**Figure 4C**). Detection of Mcl1 in DEspR^+^ [NET^+^Ns] suggests longer neutrophilic lifespans, as observed in DEspR^+^ neutrophils (20), hence delaying clearance by apoptosis-dependent efferocytosis (34, 35).

### Detection of EPCAM^+^ circulating tumor cells (CTCs) with NET^+^N ‘escorts’

**Figure 5:**
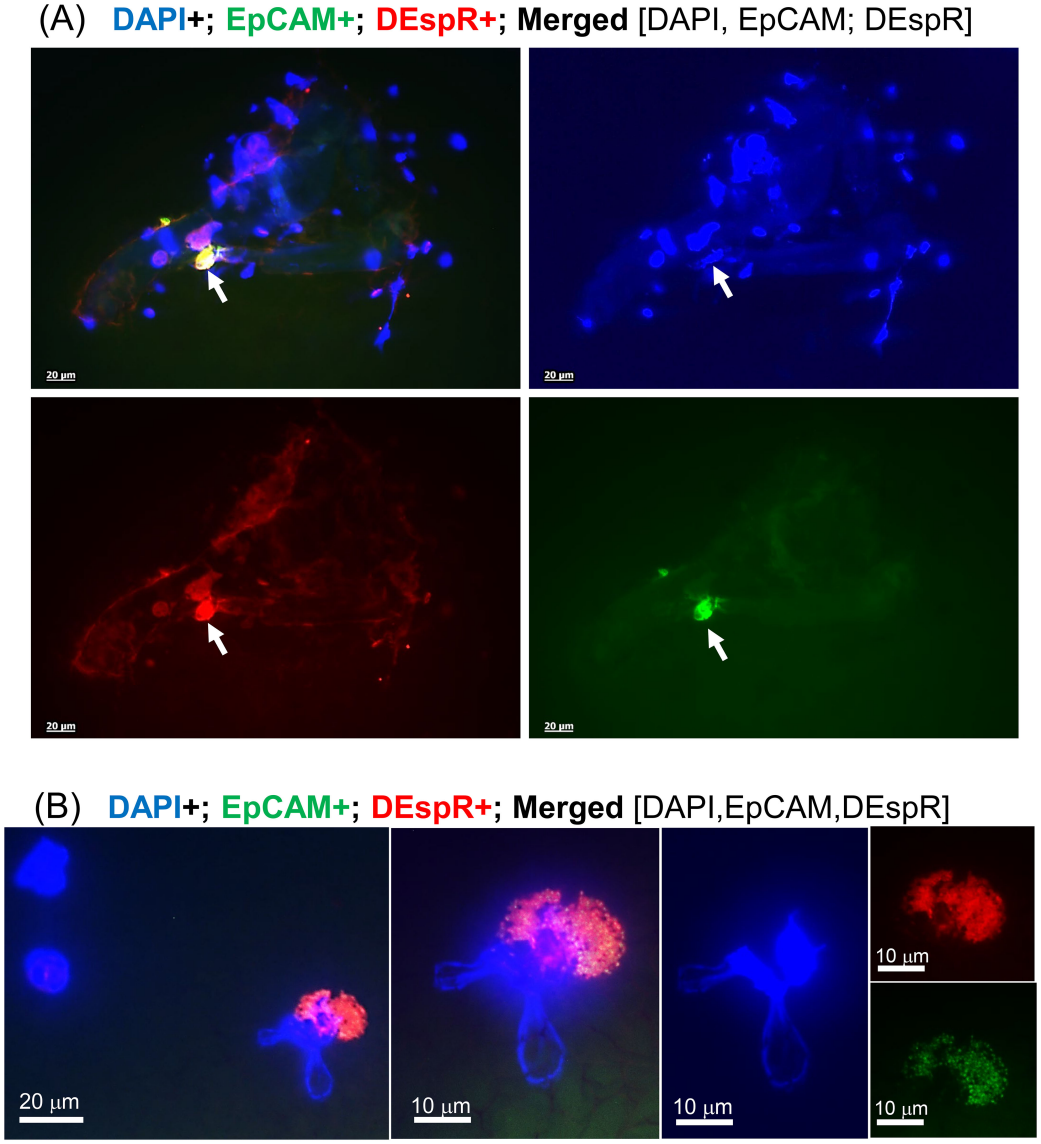
Representative images of DEspR+/EpCAM+ circulating tumor cells **(CTCs)** complexed with [NET^+^Ns] at baseline. **(A)** IFC analysis of 5μL buffy coat (mNSCLC 6m OS) detects EpCAM+DEspR+ CTC (↑) entangled within a cluster of [NET^+^Ns]. Higher DEspR expression in EpCAM+CTC than in adjacent [NET^+^N] (EpCAM[-]). Bar = 20 μm. **(B)** IFC analysis of 5 μL buffy coat (mNSCLC 3m OS) detects DEspR+EpCAM+ CTC-complexed to NET’s extruded DNA. Two DEspR[-]EpCAM[-] cells as contrast depict IF-staining specificity for DEspR+EpCAM+ expression. Bar = 20 μm, left panel; bar = 10 μm, rest of image panels. Two CTCs detected in both 5 μL samples; no DEspR[-] EpCAM+ CTCs observed.

To determine whether DEspR^+^ [NET^+^Ns] interact with circulating tumor cells (CTCs) similar to neutrophils (36), we performed IFC using DEspR and EPCAM dual antibody staining. As shown in **Figure 5**, IFC detected EPCAM^+^ CTCs within DEspR^+^[NET^+^N] clusters (**Figure 5A**) or with a solitary DEspR^+^[NET^+^N] with extruded NET (**Figure 5B**). Interestingly, relative expression levels of DEspR were greater in CTCs than in NET^+^Ns (**Figure 5A**). We note that not all NSCLC patient samples exhibited NET^+^N/CTC-complexes. However, testing was limited to a randomly selected 5 μL of buffy coat sample prepared from 10 mL of EDTA-anticoagulated fresh whole blood.

### Corroboration of circulating NET^+^Ns and complexes by flow cytometry

**Figure 6:**
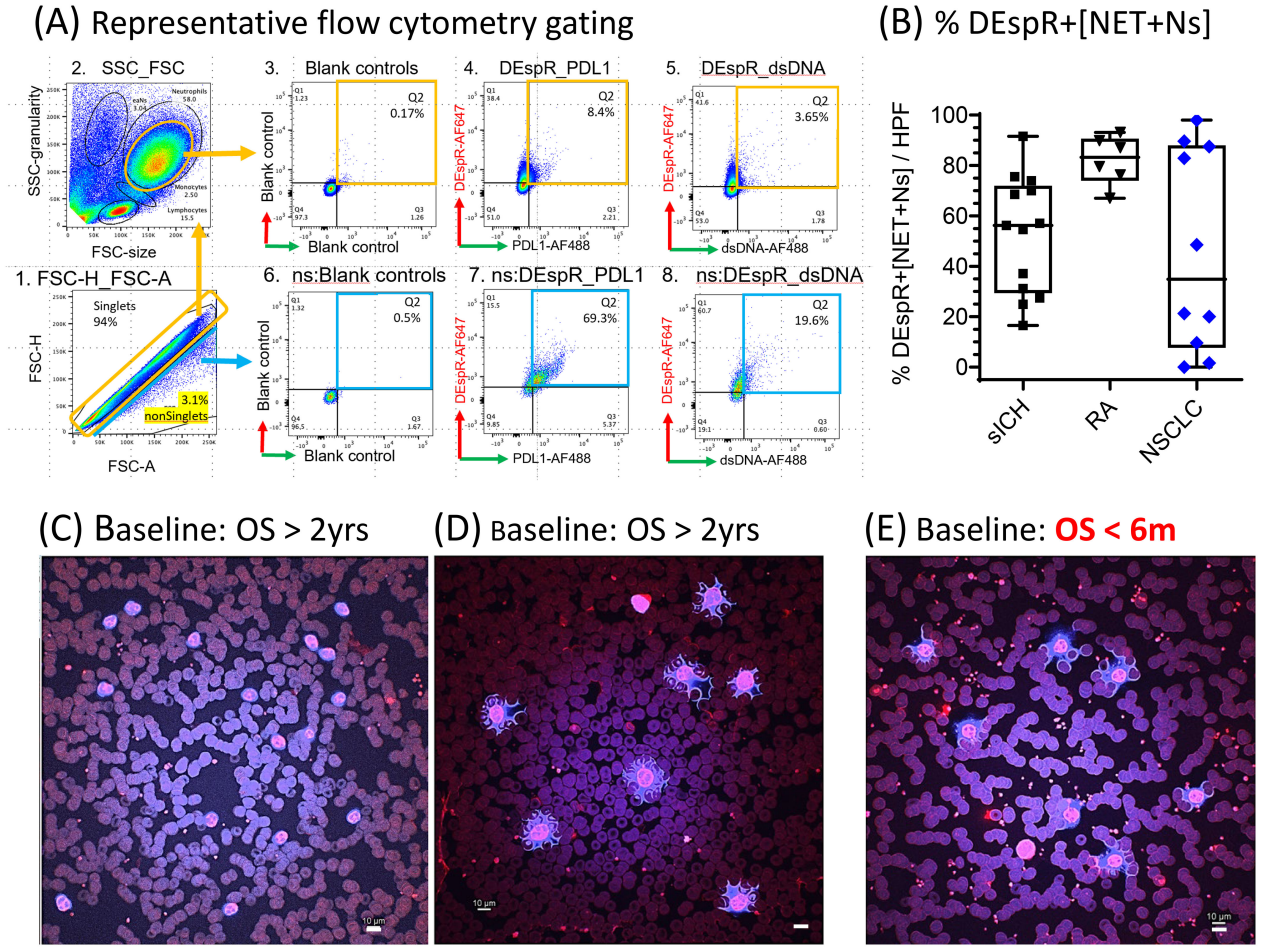
Baseline circulating levels of DEspR+ [NET^+^N] subtype and microvesicles in mNSCLC. **(A)** Representative flow cytometry gating panels #1-8: 1] gating for selection of singlet cells vs non-singlet cells (doublets, clusters < 35 microns) to confirm observed [NET^+^N] doublets [NET^+^N]-RBC clusters; 2] typical FSC/SSC gating for neutrophils; 3] quadrant analysis for background in quadrant 2 (Q2); 4] Q2 analysis for dual staining of DEspR+PDL1+ neutrophil subtype; 5] Q2 analysis for DEspR+dsDNA+ on neutrophil surface, thus indicating extruded DNA; 6] Q2 demonstration of background from blank controls in non-singlets (ns); 7] Q2 demonstration of high %DEspR+PDL1+ among non-singlets; 8] Q2 depicts DEspR+dsDNA+ non-singlets. **(B)** Comparison of % DEspR+PDL1+ neutrophils in NSCLC patients compared to published sICH [18] and RA [19] patient samples. **(C, D)** Representative confocal IFC images from an mNSCLC patient with progression-free survival (PFS) and overall survival (OS) > 2 years: low magnification to show low levels of [NET^+^Ns] and higher magnification to show low levels of microvesicles (MVs). **(E)** Representative confocal IFC image of mNSCLC patient with OS < 90 days. DEspR+ [NET^+^Ns] with many more microvesicles (netMVs) compared with panel **(D)**.

To corroborate DEspR+ [NET^+^Ns] detected by IFC in patient blood smears, we tested for double-immunostaining for DEspR and extruded double-strand DNA (dsDNA) using non-permeabilized flow cytometry. Detection of DEspR^+^dsDNA^+^ neutrophils by flow cytometry (**Figure 6A**) corroborated DEspR^+^ [NET^+^Ns]. Detection of DEspR^+^PDL1^+^ neutrophils by flow cytometry (**Figure 6A**) corroborated IFC findings of DEspR^+^PDL1^+^ neutrophils and NET^+^Ns (**Figures 3**, **4**).

To corroborate observed NET^+^N doublets and/or NET^+^N-RBC clusters observed by IFC confocal images, we analyzed the non-singlets pool by flow cytometry. Consitent with the IFC finding, as shown in **Figure 6A**, we detected DEspR^+^PDL1^+^ and DEspR^+^dsDNA^+^ non-singlets < 35 microns.

### Association of proportion (%) and number (#) of DEspR^+^ NET+Ns/HPF and DEspR^+^MVs/HPF-counts with overall survival

To identify potential parameters that meet reliable quantitation, we compared the % DEspR^+^ NET^+^Ns among all patients with mNSCLC. We detected a wide variation in the percentage of DEspR+ [NET^+^Ns] in mNSCLC (**Figure 6B**), similar to that reported in other diseases in which NETs are associated with disease exacerbation, including spontaneous intracerebral hemorrhage (sICH) and rheumatoid arthritis (RA) (**Figure 6B**). Next, we analyzed IFC results from two patients representing opposite outcomes: one mNSCLC patient with the longest PFS, OS > 2 years, and no immune-related adverse effects (**Figures 6C, D**), and one non-responder with the shortest OS (< 90 days) (**Figure 6E**). Interestingly, this revealed that the number (#) of DEspR^+^[NET^+^Ns]/HPF and the number of DEspR^+^ MV levels/HPF differentiated patients with relatively long survival from patients with shorter survival. These observations support the use of these parameters for exploratory quantitative analysis: percentage DEspR+ [NET+Ns], number of NET+Ns per HPF, and number of MVs per HPF.

**Figure 7:**
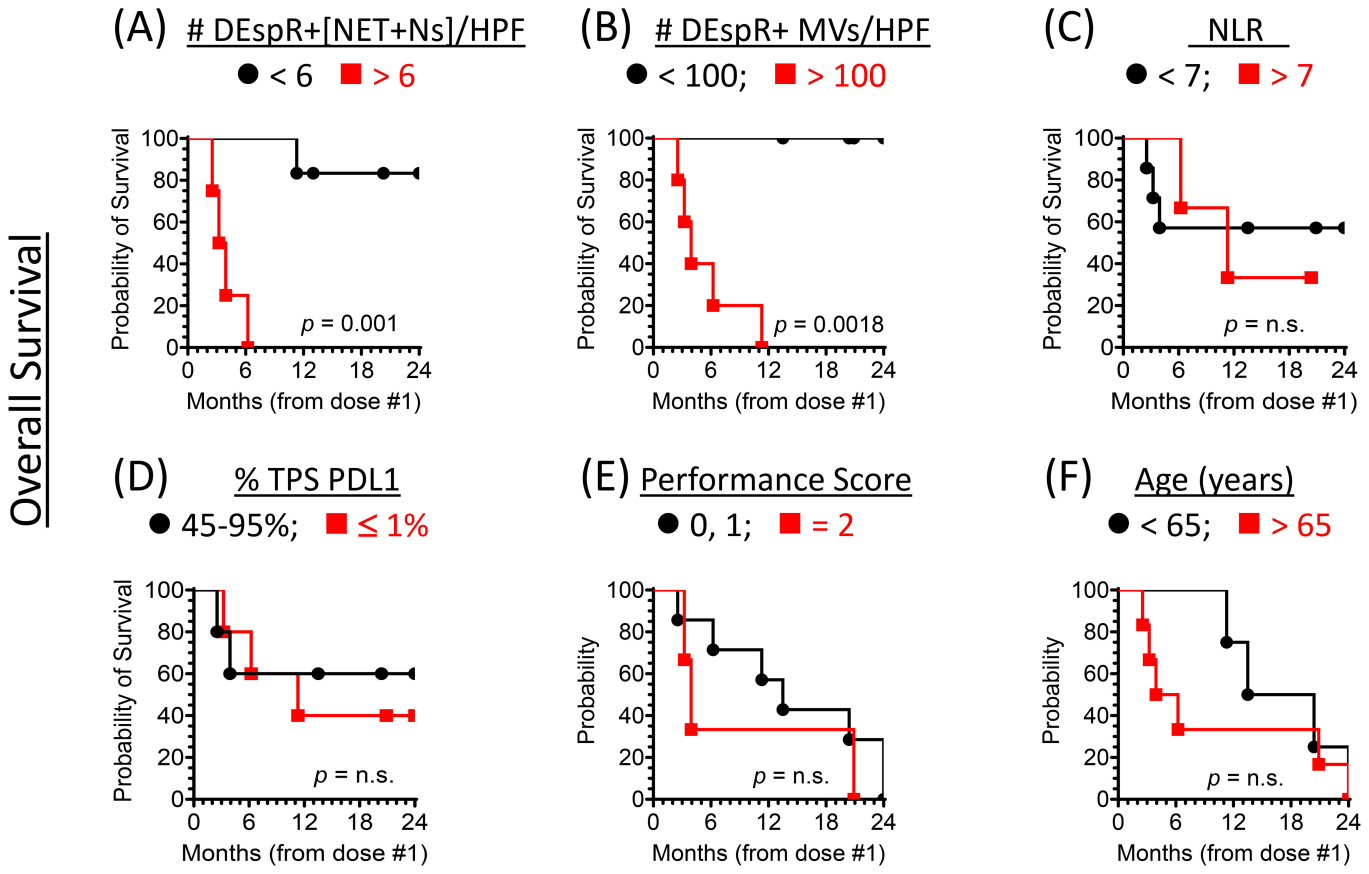
Kaplan-Meier (KM) survival curve analysis for overall survival (OS) for 8 parameters with parameter-specific thresholds. Parameters are: **(A)** Number (#)DEspR^+^[NET^+^Ns] among all neutrophils in 6 high power fields (HPF) observed, Mantel–Cox log-rank *p* < 0.001 for OS; **(B)** #DEspR+ microvesicles(MVs)/HPF, Mantel–Cox log-rank *p* < 0.0018 for OS. All other parameters tested in panels **(C–H)** exhibit no significant differences (n.s.) in survival probability (Mantel–Cox log-rank for OS p = n.s.): **C** NLR > 7 vs NLR < 7 cut-off; **(D)** % tumor proportion score (TPS) for intratumoral PDL1 (Programmed Death Ligand-1) expression ≤ 1% vs > 1%; **(E)** ECOG (Eastern Cooperative Oncology Group) Performance Score 0,1 vs 2; **(F)** age ≥ 65 vs < 65 years.

We therefore performed comparative Kaplan–Meir (K–M) analyses. (**Figures 7A–F**). Using a cutoff threshold of 50% for the proportion (%) of DEspR^+^[NET^+^Ns] among all neutrophils/HPF, Kaplan–Meier survival curve analysis detected a borderline difference (data not shown). Using a cutoff threshold of 6 for #DEspR+ [NET+Ns], K–M analysis showed significant differences (p < 0.001) (**Figure 7A**). Using a threshold of > 100 for MVs/HPF, K–M survival curve analysis showed significant differences (p = 0.0018) (**Figure 7B**). In contrast, no cutoff thresholds significantly distinguished OS (or PFS; data not shown) for NLR, %PDL1-TPS, ECOG performance status, and age (p = n.s. for all; **Figures 7C–F**).

Consistent with these findings, exploratory Cox regression with penalized Firth’s likelihood for small sample size and Benjamini–Hochberg procedure to reduce false discovery rate (21, 22) detected significant association of DEspR^+^ MVs/HPF counts and percentage DEspR^+^ [NET^+^Ns] with OS, with a borderline trend for #DEspR^+^ [NET^+^N] counts, in contrast to no substantial association trend for NLR, % PDL1-TPS, age, ECOG performance status, sex, and treatment regimen with or without radiation therapy (**Table 1**).

**Table 1:** Variables tested with univariate Cox regression analysis with Firth’s penalized likelihood for association with overall survival.

Variables ^†^	Cutoff [median]	HR (95% CI)	Unadjusted *p*-value	Adjusted *p*-value B-H ^§^
#DEspR+[NET+Ns]/HPF	≤5 (Ref) >5	6.39 (1.08 – 67.49)	0.04 ^*^	0.15
#DEspR+[MVs]/HPF	≤100 (Ref) >100	25.61 (2.65 – 3,439.14)	0.0025 ^**^	0.01 ^*^
%DEspR+[NET+Ns]	≤50% (Ref) >50%	28.6 (2.8 – 3873.97)	0.0026 ^**^	0.01 ^*^
NLR	≤7 (Ref) >7	1.31 (0.22 – 6.76)	0.75	0.92
%PDL1 TPS	≤1%(Ref) >1%	0.79 (0.13 – 4.07)	0.77	0.92
Age (years)	Continuous	1.01 (0.93 – 1.09)	0.85	0.92
Sex	Female (Ref) Male	0.92 (0.18 – 5.55)	0.92	0.92
Cancer Stage	IIIA/IVA (Ref) IVB	3.61 (0.65 – 36.58)	0.15	0.29
ECOG	0/1 (Ref) 2	2.34 (0.38 – 12.56)	0.33	0.48
Treatment Regimen	Chemo-IO RT, chemo-IO	0.30 (0.03 – 1.66)	0.17	0.29

n = 10 for all variables, Ref: reference group, HR: hazard ratio, CI: confidence interval, ^†^variables obtained pretreatment with chemo-IO, ^§^Benjamini–Hochberg procedure to control for false positive rates, ^*^p-value < 0.05, ^**^p-value < 0.01; variables tested: #DEspR^+^[NET^+^Ns]/HPF, average counts of NET^+^Ns across six HPFs (60x confocal microscopy of IF-cytology DESpR and citH3 staining to confirm NET^+^Ns); #DEspR^+^[MVs/HPF], the average number of DEspR+ microvesicles released by DEspR^+^ [NET^+^Ns] per HPF across six random HPFs on IF-cytology; %DEspR^+^[NET^+^Ns], proportion of DEspR^+^ neutrophils extruding NETs among all cells across six HPFs on IF-cytology; NLR, neutrophil-lymphocyte ratio; %PDL1 TPS, % expression of PDL1 (programmed death receptor ligand-1) tumor proportion score; ECOG performance score, ECOG, Eastern Consortium Oncology Group performance score. Treatment regimen: chemo-IO, combination immune checkpoint inhibitor with chemotherapy; RT, chemo-IO, radiation therapy prior to chemo-IO; TPS, tumor proportion score. Bold font, highlight significant p values.

To address the 1-year survival rate of 31% in mNSCLC, RMST analysis (**Table 2**) was performed. Patients with higher #DEspR^+^ [NET^+^N]/HPF levels (> 5) had an average survival of 5.6 months, compared with 11.9 months for patients with lower levels (≤ 5) within a 12-month observation period. This represents a statistically significant 52.9% (1–0.471) reduction in 1-year survival time associated with elevated #DEspR^+^[NET^+^N]/HPF levels. Similarly, patients with higher #DEspR^+^[MVs]/HPF levels (> 100) had an average survival of 5.44 months, compared with 12 months for patients with MV levels ≤ 100 within a 12-month observation period. This represents a statistically significant 54.6% (1–0.454) reduction in 1-year survival time associated with elevated #DEspR+[MVs]/HPF levels. Finally, patients with higher %DEspR^+^ [NET^+^N] levels (> 50) had an average survival of 3.98 months, compared with 11.88 months for patients with lower levels (≤ 50) within a 12-month observation period. This represents a statistically significant 66.5% (1–0.335) reduction in 1-year survival time associated with elevated %DEspR^+^[NET^+^N] levels.

**Table 2:** One-year restricted mean survival time (RMST) estimates, differences and ratios.

1-year endpoint:➔ ↓variables:	RMST estimate arm 1	RMST estimate arm 0	RMST difference (estimate 95% CI), *p*-value	RMST ratio (95% CI) *p*-value
#DEspR^+^[NET^+^Ns]/HPF	> 5 RMST = 5.6 m	< 5 RMST = 11.9 m	–6.276 m (–9.302, –3.25) *p* < 0.001 ***	0.471 (0.274, 0.808) *p* = 0.006 **
#DEspR^+^[MVs]/HPF	> 100 RMST = 5.4m	≤ 100 RMST = 12.0 m	–6.556 m (–9.344, –3.768) *p* < 0.001 ***	0.454 (0.272, 0.757) *p* = 0.002 **
%DEspR^+^[NET^+^Ns]/HPF	> 50 RMST = 4.0 m	≤ 50 RMST = 11.9 m	–7.903 m (–9.282, –6.525) *p* < 0.001 ***	0.335 (0.238, 0.472) *p* < 0.001 ***

Arms 1,0: groups distinguished using median as cutoff; #DEspR^+^[NET^+^Ns]/HPF, average number (counts) of DEspR^+^NET-forming neutrophils per high power field (HPF) across six 60x-oil confocal microscopy HPFs; #DEspR^+^[MVs]/HPF, average number of DEspR^+^(citH3^+^) microvesicles released by NET-forming neutrophils per HPF across six 60x-oil confocal microscopy HPFs; %DEspR^+^[NET^+^Ns]/HPF, proportion (%) of DEspR^+^ NET-forming neutrophils among all cells in six 60x-oil confocal microscopy HPFs; CI, 95% confidence interval; m, months. RMST, restricted mean survival time; RMST estimate, average survival time per Arm within 1-year study cutoff (τ); RMST difference, difference between Arm1 *vs* Arm2 areas under the survival curves quantifying the average postponement of death within the 1-year specified cut-off; RMST ratio, Arm1/Arm0 ratio of 1-year RMST estimates quantifying how much shorter Arm 1 subjects (with higher # or % DEspR^+^[NET^+^N] or MV measures) are expected to survive compared with Arm 0 (with less # or % DESpR^+^[NET^+^N] or MV measures. The RMST was analyzed in R (4.5.0) using the “survRM2” package **, p < 0.01, ***, p < 0.001.

Exploratory univariate linear regression for 1-year OS demonstrated significant adjusted R^2^ values for #DEspR^+^MVs/HPF and for both the number and percentage of DEspR^+^[NET^+^Ns]/HPF (**Table 3**), giving a basis for a larger study to test for multivariate analyses.

**Table 3:** Univariate linear regression analysis using the 1-year survival study endpoint.

Variables	Coefficient	R^2^	Adjusted R^2^	*P* value	Normality	Power
#DEspR+[NET+Ns]/HPF	–0.9521	0.755	0.720	0.0024	Passed (P = 0.391)	0.902
#DEspR+MVs/HPF	–0.0537	0.788	0.757	0.0014	Passed (P = 0.654)	0.932
%DEspR+[NET+Ns]/HPF	–0.0946	0.818	0.792	0.0008	Passed (P = 0.194)	0.956

#DEspR^+^[NET^+^Ns], average number (counts) of DEspR^+^ NET-forming neutrophils per high power field (HPF) across six 60x-oil confocal microscopy HPFs; #DEspR^+^[MVs], average number of DEspR^+^citH3^+^ microvesicles released by NET-forming neutrophils per HPF across six 60x-oil confocal microscopy HPFs; %DEspR^+^ [NET^+^Ns], proportion (%) of DEspR^+^NET-forming neutrophils among all cells in six HPFs from 60x-oil confocal microscopy; HPF, high power field from 60x-oil confocal microscopy imaging; MVs, microvesicles; [NET^+^Ns], NET-forming neutrophils; R^2^, R-squared or the coefficient of determination; adjusted R^2^, R^2^ penalized for sample size in univariate linear regression to prevent overfitting and overestimation of explanatory power.

### DEspR^+^PDL1+ co-expression in lymph node metastasis and primary NSCLC tumor

**Figure 8:**
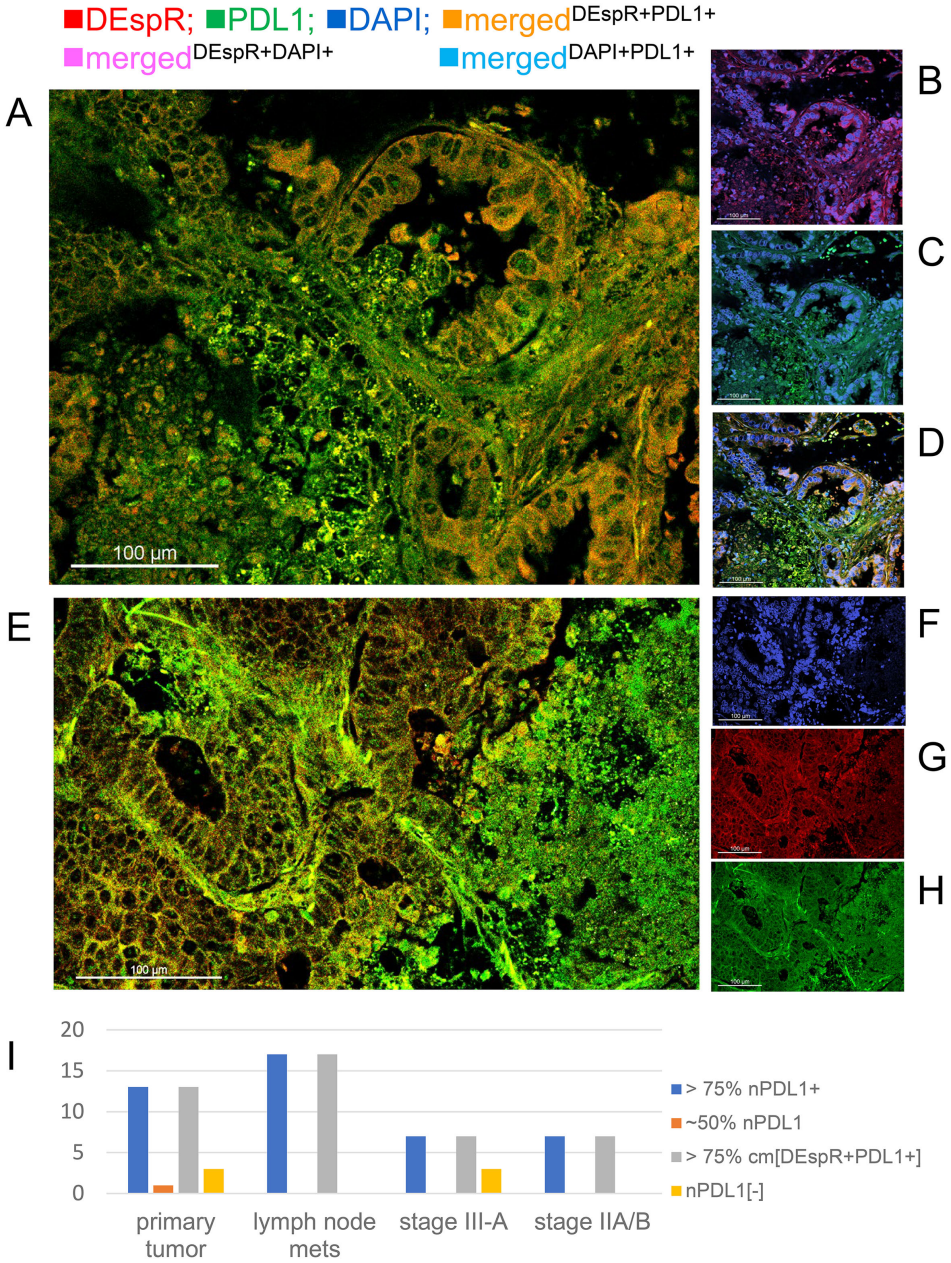
Representative EDF confocal composite images of 0.25 micron z-stacks of lymph node metastasis **(A–D)** and its corresponding primary NSCLC adenocarcinoma, T3N1M0, Stage III-A **(E–H)**. A merged immunofluorescence image of lymph node (LN) metastasis showing DEspR (red) and PDL1 (green) cell membrane and cytoplasmic co-expression (merged orange) in tumor cells, inflammatory cells, and microvesicles, and nuclear localization of PDL1 in metastatic tumor cells, **(B)** merged DEspR+DAPI+, **(C)** PDL1+DAPI+, and **(D)** merged DEspR, PDL1+, DAPI. **(E)** Merged DEspR+PDL1+ image of primary NSCLC adenocarcinoma (T3N2M0) Stage III-A; **(F)** DAPI, **(G)** DEspR, **(H)** PDL1. Bar = 100 microns. **(I)** Analysis of IF-staining of nuclear PDL1 (nPDL1) expression in 17 NSCLC adenocarcinoma primary tumors and corresponding lymph node (LN) metastases cores (n = 17 adenocarcinoma NSCLC, stage III-A and stage II-A, with nuclear grade 2-3). Nuclear PDL1^+^DEspR^+^ expression was detected in tumor cells in all 17 lymph node metastasis, but in 14 of 17 primary NSCLC adenoca tumors. Majority of tumor cells exhibited ≥ 75% DEspR^+^PDL1^+^.

To test whether DEspR+PDL1+ co-expression is detected in mNSCLC, we analyzed matched primary NSCLC and corresponding lymph node metastases (**Figure 8**). In mNSCLC lymph node metastases, immunofluorescence detected DEspR+PDL1+ co-expression in the cell membranes of metastatic tumor cells and inflammatory cells and detected MVs in the adjacent interstitium and in sites of cell necrosis (**Figures 8A–D**). Nuclear PDL1+ expression was also observed in metastatic tumor cells in lymph nodes (**Figures 8A–D**). Consistent with these findings, similar tumor cell findings were detected in the corresponding NSCLC primary tumors (**Figures 8E–H**).

To determine the extent of DEspR^+^nPDL1^+^ co-expression, we analyzed an NSCLC tumor array comprised of cores isolated from the primary NSCLC adenocarcinoma tumor (n = 17, stage II-A/B and stage III-A, and nuclear grades 2–3) and corresponding lymph node metastases (n = 17) by dual IF-staining for DEspR and PDL1. Confocal multiplex fluorescence microscopy showed nuclear PDL1 in DEspR^+^ tumor cells in 14 of 17 tumor cores and in all 17 lymph node metastases. Approximately 75% of tumor cells were DEspR^+^nPDL1^+^ (**Figure 8I**).

### Corroboration of the DEspR-specific transcript in RNA-seq datafiles

To strengthen these findings at the protein level, we next corroborated the DEspR-specific spliced transcript with the trp#14 codon, which is distinguished from the non-spliced FBXW7-AS1 non-coding RNA with a stop codon (**Supplementary Figure 3A**). Human RNA-seq files were analyzed using a stepwise sample channel staging approach, restricting the analysis to confirmed human-specific sequences (**Supplementary Figure 3B**). This revealed DEspR-specific RNA transcripts predominantly in cancer samples (**Supplementary Figure 3B**). Notably, the DEspR-specific transcripts were annotated as FBXw7 transcripts (**Supplementary Figure 3B**), an error likely preceding the recognition of the DEspR protein (UniProt.org) but now requiring correction in NCBI databases.

## Discussion

### Insights into immunosuppressive PDL1^+^ neutrophils and NET+N subtypes

The detection of NET^+^Ns in different stages of NET formation indicates an intravascular microenvironment that sustains ongoing NET formation of neutrophils released daily at an estimated rate of 10^9^ neutrophils per kg per day (37). The detection of all steps of NET formation (extruded DNA with citH3^+^DNA in the cytoplasm, recognizable polylobulated nuclei with decondensed or rounded nuclei, and focal cell membrane microruptures) at the same time in IFC images contrasts with the ordered sequential steps of NET formation observed *in vitro* following stimulation with *Candida albicans* (early MV release, followed by nuclear decondensation and rounding, then nuclear envelope rupture, and finally cell membrane rupture and expulsion of DNA) (38). These differences could be due to different NET-formation stimuli. Nevertheless, they highlight the importance of studying molecular-cellular features by direct visualization and levels of circulating NET^+^Ns in blood samples obtained from patients with mNSCLC at disease- and treatment-specific time points.

The direct visualization of DEspR^+^ [NET^+^Ns] interconnections with CTCs and RBCs in IFC images provides translational support for emerging pathogenic concepts in cancer progression. CTC-[NET^+^N] complexes in patients with mNSCLC provide translational support for the concept of NETs as scaffolds or shields on CTCs that promote CTC-immune evasion and metastasis competence (10, 12, 39). RBC[NET^+^N] complexes indicate the likelihood of low-flow ischemia, providing translational support for observations from mouse models that ischemia-mediated inflammatory responses may contribute to cancer progression (40).

The association between increased DEspR^+^PDL1^+^ MVs and decreased overall survival is consistent with reports that PDL1^+^ extracellular vesicles (EVs), such as exosomes and microvesicles, are associated with intrinsic and acquired ICI-resistance, immune evasion, and metastasis (27, 41). While PDL1^+^ EVs have typically been attributed to release by tumor cells, IFC imaging demonstrated circulating DEspR^+^PDL1^+^citH3^+^ MVs released from circulating NET^+^Ns, consistent with MV release as a step in NET formation (or NETosis) (38). Importantly, DEspR^+^PDL1^+^ [NET^+^Ns] as a source of PDL1^+^ MVs suggests an additional contributor to the total PDL1 burden and may help explain the efficacy of ICIs in patients whose tumors are PDL1-negative (42). These data support the emerging concept that systemic PDL1 burden, in addition to the tumor microenvironment (TME), may influence responses to immunotherapy (13). 

### Translational implications

Although this was a small pilot study, the observation of cutoff values associated with significant differences in OS in K–M-analysis, supported by Cox regression analysis, RMST, and linear regression, supports further evaluation of the number and proportion of DEspR^+^[NET^+^Ns] and the number of DEspR^+^ MVs as potential biomarkers of < 1-year OS in larger, independent cohorts of patients with mNSCLC receiving first-line chemo-IO and with ECOG performance scores 0–2. While confirmatory studies remain to be done, the observed associations reported here are supported by converging evidence from our previous studies of DEspR+ neutrophils and tumor cells, together with studies by others investigating the biology of NET formation and the “double-edged sword” roles of neutrophils and NET^+^Ns in pathogen defense on one hand and tissue injury and cancer metastasis on the other.

This converging evidence spans different research approaches. First, DEspR^+^ neutrophils and NET^+^N counts have also been associated with poor survival outcomes in patients with acute spontaneous intracerebral hemorrhage (sICH) (18). In animal models, targeted DEspR inhibition via multiple blocking anti-DEspR antibodies reduced poor outcomes *in vivo* in different sterile inflammation models, including LPS-induced rat encephalopathy, macaque LPS-induced transient acute lung injury models (20), and acute sICH in a rat model (43). Additionally, the observed persistence of DEspR^+^PDL1^+^ neutrophils, NET^+^Ns, and their MVs 6 weeks after the first dose of chemo-IO is consistent with the previously reported apoptosis resistance and longer lifespans of DEspR^+^ neutrophils compared with DEspR^[-]^ neutrophils (17). Furthermore, co-expression of DEspR^+^ cell membranes with nuclear PDL1^+^ tumor cells in mNSCLC primary and lymph node metastases is consistent with DEspR roles in cancer stem cell (CSC) pro-metastasis anoikis resistance (33) and with nuclear-PDL1 roles in CSC-stemness (44), immune evasion (45), and metastasis (46). The detection of approximately 1 micron microvesicles emerging from NET^+^Ns, with some still embedded within the extruded NET DNA, is consistent with reported live-cell imaging studies demonstrating microvesicle release in NET formation/NETosis (38). Finally, the observation of circulating DEspR^+^EpCAM^+^ CTCs wrapped by the extruded DNA of DEspR^+^[NET^+^Ns] is consistent with reports that NETs trap lung tumor cells and promote liver metastasis in mouse models (47).

This converging evidence, along with our current data, provides a basis for evaluating DEspR+ neutrophils and NET+Ns in larger, independent cohorts as potential mechanism-based biomarkers that are targetable on DEspR^+^PDL1^+^ neutrophils and on DEspR^+^nuclearPDL1^+^ tumor cells. The potential to selectively preempt NET formation and MV release only in pathogenic DEspR^+^ neutrophils able to resist chemotherapy becomes a potential approach that avoids loss of pathogen defense by sparing all DEspR[-] neutrophils and NET^+^Ns for homeostatic immune surveillance. Neutrophil subset-specific targeting is critical as non-specific inhibition of neutrophil adhesion has previously been associated with an increased incidence of major infections (48).

These observations at the protein level are strengthened by the corroboration of DEspR-specific transcripts in human RNA-seq files. Notably, DEspR-specific transcripts are erroneously assigned to the larger FBXw7 transcript on the DEspR’s antisense strand and may require updated annotation. The detection of both the non-coding antisense FBXw7-AS1 and the coding DEspR-specific RNA indicates a bifunctional coding and non-coding RNA (cncRNA), as defined previously (49).

## Conclusions

DEspR^+^ expression marks a subtype of: a] PDL1^+^ neutrophils and monocytes, b] citH3^+^[NET^+^Ns] and MVs, and c] [NET^+^N]-epCAM^+^CTC complexes. Data identify DEspR^+^ [NET^+^N] subtype levels as potential pretreatment biomarkers of poor 1-year OS in patients with mNSCLC receiving first-line chemo-IO. Collectively, these findings identify a targetable neutrophil and NET^+^N subset in mNSCLC and provide a basis for further studies of neutrophil and NET^+^N subtyping in larger, independent cohorts of patients with mNSCLC.

### Study limitations

This prospective observational study is limited by its small sample size. However, detection of increased and persistent DEspR^+^PDL1^+^/citH3^+^ NET^+^Ns and DEspR^+^PDL1^+^/citH3^+^ MVs in sterile inflammation, together with detection of DEspR^+^nuclearPDL1^+^ tumor cells in primary and lymph node metastasis in mNSCLC, represents novel findings that are not dependent on the number of patients. Additionally, while direct visualization methodology provides novel insights into molecular subtyping and cell-cell complexes of patient-specific circulating NET^+^Ns, allows semi-quantitation of NET^+^Ns and MVs, and sets a standard over indirect measurements of myeloperoxidase-DNA complexes and citH3 plasma levels, the direct visualization methodology needs improvement for faster applications.

## Data Availability

The original contributions presented in the study are included in the article/**Supplementary Material**. Further inquiries can be directed to the corresponding author.

## Supplementary material

Supplementary Figure 1 Representative images of [NET^+^Ns] and [NET^+^N] interactions captured in 5 μL fresh whole blood smear. **(A, B)** Two distinct regions of interest (ROI) on the same cytology slide show multiple [NET^+^Ns] in panel A, and no [NET^+^Ns] in panel B, thus ascertaining that detection of extruded DNA (DAPI+ blue) in DEspR+ neutrophils (magenta) is not an artifact of cytology slide preparation or IF-staining. **(C, D)** Representative ROIs depicting multiple [NET^+^N] with NET-DNA forming stringy DNA-interconnections. Red, DEspR+; Blue, DAPI+ DNA. Bar, 10 microns.

Supplementary Figure 2 One-micron microvesicles released from circulating NET+Ns. **(A)** Multiple NET+Ns (#1-#4) are DEspR+. (➔) monocyte with no extruding NETs. **(B)** DAPI+ staining shows extruded DNA wrapping around RBCs and some DNA+DEspR+ microvesicles. **(C)** DEspR+ [monocyte-NET^+^N] in close proximity to a [NET^+^N] with DEspR+ microvesicles. Bar = 10 microns.

Supplementary Figure 3 Identification of DEspR-specific transcripts in open-access RNA-seq datafiles. **(A)** Diagram of DEspR-specific RNA transcript sequence used to search RNA-seq data files. The DEspR-specific transcript sequence is comprised of spliced DEspR exon-1 and exon-2, and contains the trp#14 codon. This is distinguished from the FBXw7-AS1 transcript, which is non-spliced and contains a stop codon at DEspR’s trp#14 codon. **(B)** Sample funnel stages and results of analysis at each funnel stage. This structured report format ensures data quality is verified at each step, preventing artifacts from affecting biological interpretation. Analysis reveals a] DEspR-specific transcripts: Trp#14 and spliced DEspR – which were mostly in cancer samples, and with lung cancer and pancreatic ductal adenocarcinoma as the top two cancer types; b] that DEspR-specific Trp#14+ 2,329 transcripts are annotated as FBXw7 – *a misread requiring updating/correction*; and c] that Trp#14 and spliced DEspR (n = 1,059) is ~50% less than truly human Trp#14 motif-bearing transcripts (n = 2,329) which is concordant with the expectation that RNA-editing occurs before splicing (50).
