# Supplementary material for: Prospective subtyping identifies an association between high pretreatment levels of DEspR+ neutrophil extracellular trap–forming neutrophils and microvesicles during sterile inflammation with poor survival in patients with metastatic non-small cell lung cancer receiving first-line combination chemo-immunotherapy
Source: Front Immunol. 2026 Aug 5;17:1849858. doi: 10.3389/fimmu.2026.1849858 (PMC13485590; doi:10.3389/fimmu.2026.1849858)
Supplement: Supplementary file 1 [file Table1.docx]

Supplementary Table 1	Demographics and clinical data

| ID # | Sex | Age (y) | Cancer stage | Metastases  Sites [*irAE] | ECOG PS | Treatment regimen | Best Response | PFS (m) | OS (m) | NLR | % TPS PDL1 | # DEspR+  NET^+^Ns | % DEspR+  NET^+^Ns | # DEspR+ MVs |
| --- | --- | --- | --- | --- | --- | --- | --- | --- | --- | --- | --- | --- | --- | --- |
| **1** | M | 63 | IV | Brain | 1 | RT, 1,2, 3 | PR | >24 | > 24 | 6.73 | 1 | 0.2 | 96.3 | 45.8 |
| **2** | M | 55 | IVB | Bone | 1 | RT, 1,2,3 | NR | 1.4 | 11.30 | 9.33 | <1% | 1 | 6.7 | 113 |
| **3** | F | 84 | IVA | Pleura [*pn] | 1 | 1 | PR | 5.0 | > 24 | 4.07 | 60 | 0 | 0 | 85.5 |
| **4** | M | 70 | IVA | Pleura | 1 | 1,2,3 | MR | 2.53 | 2.53 | 2.1 | 50 | 8.6 | 92 | 162.2 |
| **5** | F | 72 | IV | Adr., bone, liver, LN, lung | 1 | 1,2,4 | PR | 2.43 | 6.23 | 9.76 | 1 | 8.5 | 94 | 154.75 |
| **6** | M | 71 | IVA | LN [*pn] | 2 | 1,2,3 | NR | 1.4 | > 24 | 6.89 | 1 | 1.67 | 31.6 | 18.67 |
| **7** | M | 47 | IVB | Brain, pleura | 1 | RT, 1,2,3 | MR | 6.3 | > 13 | 7.62 | 95 | 1.2 | 15 | 27.6 |
| **8** | F | 64 | IIIA | Ipsi LN [*hep] | 0 | RT, 2,5,6 | PR | 2.0 | > 20 | 0.9 | 60 | 5.17 | 77.8 | 41.33 |
| **9** | M | 66 | IVB | Liver | 2 | 1,2,3 | NR | 3.0 | 3.93 | 3.89 | 45 | 7 | 89 | 194.6 |
| **10** | F | 67 | IVB | Liver | 2 | 1,2,3 | NR | 2.5 | 3.23 | 6.55 | <1% | 6.83 | 83.7 | 110.5 |

Chemotherapy-IO combination treatment regimen: #1, #2, #3: 1] Pembrolizumab, 2] Carboplatin, 3] Pemetrexed, 4] Abraxane, 5] Durvalumab, 6] Paclitaxel; Best response: CR, complete response; PR, partial response; MR, mixed response or steady disease; NR, no response; #DEspR^+^[NET+Ns], average number of DEspR^+^ [NET^+^Ns] per high power field of view (HPFs) n = 6 [600X confocal HPF of view]; %DEspR^+^[NET^+^Ns], average proportion of [NET^+^Ns], n = 6 HPFs, that are DEspR^+^; # DEspR^+^ MVs, average number of DEspR^+^ microvesicles (1 micron diameter) per HPF (n = 6 HPFs); ECOG-PS, performance status score Eastern Cooperative Oncology Group ECOG; Adr, adrenal gland; F, female; *hep, hepatitis irAE; *irAE, immune related adverse event; LN, lymphnode; M, male; m, months; NLR, neutrophil-lymphocyte ratio; OS, overall survival; PDL1, program death receptor ligand-1; PFS, progression free survival; *pn, pneumonitis irAE; RT🡪, radiation therapy before ICI+ctx; % TPS-PDL1, Tumor Proportion Score for PD-L1 expression; Survival > x-months: ongoing monitoring, non-deceased at last study determination, ID #7, lost to follow-up at 13.0 months; deceased, OS overall survival in months; y, year; end of study: 24 months.
